# “I Try and Smile, I Try and Be Cheery, I Try Not to Be Pushy. I Try to Say ‘I'm Here for Help’ but I Leave Feeling… Worried”: A Qualitative Study of Perceptions of Interactions with Health Professionals by Community-Based Older Adults with Chronic Pain

**DOI:** 10.1371/journal.pone.0105450

**Published:** 2014-09-04

**Authors:** Amanda Clarke, Denis Martin, Derek Jones, Patricia Schofield, Geraldine Anthony, Paul McNamee, Denise Gray, Blair H. Smith

**Affiliations:** 1 School of Health, Community and Education Studies, Northumbria University, Newcastle, United Kingdom; 2 Health and Social Care Institute, Teesside University, Middlesbrough, United Kingdom; 3 School of Health and Social Care, University of Greenwich, London, United Kingdom; 4 Medical Research Institute, University of Dundee, Dundee, United Kingdom; 5 School of Medicine and Dentistry, University of Aberdeen, Aberdeen, United Kingdom; University of Missouri Kansas CIty School of Medicine, United States of America

## Abstract

**Background:**

Over 50% of community-dwelling older adults experience chronic pain, which threatens their quality of life. Of importance to their pain management is older people's interaction with health professionals that, if unsatisfactory, may impair the outcome.

**Aims:**

To add to the limited research specific to older people living with chronic pain in the community, we explored how they perceive their experiences of interacting with health professionals, seeking factors that might optimise these interactions.

**Methods:**

Purposive sampling was used to recruit men and women >65 years with self-reported musculoskeletal chronic pain. Qualitative individual interviews and one group interview were undertaken with 23 participants. Data were transcribed verbatim and underwent Framework Analysis.

**Results:**

Three themes were identified. *Seeking help* illustrates issues around why older people in the community may or may not seek help for chronic pain, and highlights the potential involvement of social comparison. *Importance of diagnosis* illustrates the desire for professional validation of their condition and an aversion to vague explanations based on the person's age. *Being listened to and being heard* illustrates the importance of empathic communication and understanding expectations, with due respect for the person's age.

**Conclusions:**

In common with people of all ages, an effective partnership between an older person in pain and health professionals is essential if pain is to be reported, appropriately assessed and managed, because of the subjective nature of pain and its treatment responses. For older people with pain, perception about their age, by both parties in the partnership, is an additional factor that can unnecessarily interfere with the effectiveness of this partnership. Health professionals should engage with older adults to clarify their expectations about pain and its management, which may be influenced by perceptions about age; and to encourage expression of their concerns, which may also be affected by perceptions about age.

## Introduction

Chronic non-malignant pain is associated with physical, emotional and social limitations [1] and is one of the most pervasive and costly health-care challenges globally [2]. Most of the burden of pain, however, rests on those personally affected. Older adults are particularly vulnerable: it affects more than 50% of those living in the community [3], prevalence increases with age, and its impact is compounded by age-related comorbidities [4], [5].

Various observations have been made about older adults with chronic pain, including a determination to get on with life [6], [7] believing that pain is an expected consequence of later life [8], [9] and, consequently, assuming that seeking help is not an option [10]; and their lack of awareness of effective treatment options [11]. Particular observations have been made about encounters between older adults with chronic pain and health professionals (HPs). These include older adults assuming that professionals ‘know best’ [12], [13]; negative interactions with HPs and a fear of medical treatment leading to older adults not seeking help at all [14]; a lack of knowledge on the part of HPs about pain in older age [15]; failure to assess pain and underestimation of pain in older age by both professionals and older individuals [15]; and HPs disbelieving older adults' accounts or believing that the accounts are exaggerated [16].

These observations clearly point towards problems in the interaction between older adults with chronic pain and HPs, problems that can affect their pain management and quality of life [17]. Such problems with interactions with health professionals are not in themselves necessarily unique to older people [18]. Also, any consideration of older people as a distinct group needs to acknowledge that age is a continuous variable with no obvious point at which populations become discretely separate. However, there are strong arguments that there are aspects of living with pain particularly shaped by living with advanced years [19].

Most of this knowledge is based on qualitative methods of enquiry, which is entirely appropriate given the match between such methods and the nature of the research questions, which focus on people's perceptions, experiences and opinions. Qualitative methods are, by nature, constrained in their ability to allow their findings to be transferred or extrapolated outwith the sample studied. Given the relatively limited amount of research in this area there is a need for more such studies to iteratively add to the knowledge being generated. Also, compared to studies on samples of older people in specialist care from pain clinics there has been little contribution to the knowledge base from studies in which samples are older people living in the community [20]. The aim of this paper, therefore, is to gain more insight into how older adults living with chronic pain in the community (and not attending pain clinics) perceive their encounters with healthcare professionals, with a view to informing and improving these interactions. To do this, we draw on data from a qualitative exploration of community-dwelling older adults' experiences of living with chronic pain.

## Methods

### Setting, recruitment and participants

Men and women were recruited from the community through media advertising and contacts with groups and organisations in North-East Scotland. Despite rigorous efforts to recruit individuals from Black and Minority Ethnic (BME) groups, the only BME individuals who opted into the study were seven people from a Chinese community group. Using purposive sampling we included all those who volunteered to participate if they were aged 65 or over, had self-reported musculoskeletal chronic pain for three months or longer, and could give informed consent.

### Data collection

In a qualitative design we aimed to undertake two in-depth interviews with each participant (four to six weeks apart) using a topic guide (Table 1) based on the literature and discussions with our advisory group. The first interview focused on day-to-day living with pain. The second interview focused on the individual in order to follow-up and clarify emerging themes.

**Table 1:** Interview Topic Guide.

Questions	Probing questions
Tell me about your pain?	What is the cause of your pain?
	How long have you had the pain?
	How would you describe your pain?
	Have you experienced pain before?
	Has the cause of your pain been diagnosed?
	Who provided the diagnosis?
	How do you feel about the diagnosis?
	Is your pain comparable to anything else?
	What is your first memory of pain?
Can you tell what it's like to live with pain?	What was your life like before the pain started?
	What effect has pain had on your life?
	How does pain make you feel?
	Do you think that getting older has affected your pain at all?
	Do you think your pain has affected the way you have aged?
What treatments have you been offered for pain?	Who offered the treatment?
	Is there anything else that you feel might help your pain?
What helps to relieve your pain?	What about prescribed medicine?
	What about complementary therapy?
	What about other?
What do you know about chronic pain?	Where did you find out this information?
	How helpful was information you received?
	If you wanted to find out more, how would you do that/who would you ask?
Any other comments?	

Out of the 23 participants recruited, 14 agreed to be interviewed twice. The nine who were interviewed only once comprised the seven Chinese participants, who declined to participate further, and two other men who were unable to participate further due to ill-health. Individual interviews were undertaken in participants' homes with all but the Chinese participants who preferred to participate together in a group interview at a community group. Although this diverted from our intention to conduct individual interviews, it was consistent with qualitative approaches, which aim to respond flexibly and sensitively to participants' needs in order to capture a range of experiences and views [21]. A worker at the Chinese community group acted as interpreter when needed. The group interview followed recommendations that respondents are questioned simultaneously for individual responses, rather than the dynamic, interactive responses of focus groups, with the emphasis on individual, rather than collective responses [21]. Each interview lasted approximately an hour, was digitally recorded with permission, and transcribed.

The interviews included general questions such as the opening question “Tell me about your pain”, with subsequent probes such as “How do you feel other people respond about your pain?” A number of distinct overarching themes emerged including how people describe pain, their views on its assessment, and experiences of interactions with health professionals. The findings on describing pain and its assessment are reported elsewhere [22]. This paper focuses on the interactions with health professionals.

### Analysis

To facilitate transparent data analysis amongst the research team, we were guided by the matrix-based principles of Framework Analysis, which involves a structured process of coding and sorting the transcribed data according to key issues and themes [23].Transcripts were read, re-read and coded using QSR NVivo version 9 to assist with sorting and retrieval of data. Codes were applied to the text independently by DG and AC.

The research team and advisory group (which included lay advisers) were consulted to enhance balanced interpretation and rigorous analysis of data. Divergent interpretations were discussed until a common thematic framework was agreed, which was then systematically applied to each transcript by DG (whilst being open to incorporating new themes). We moved back and forth between the full interview transcripts and the thematic framework to ensure that interpretations were valid and contextualised in participants' broader accounts. Table 2 shows the thematic chart with examples of its application to one transcript.

**Table 2:** Example of Thematic Chart.

*Seeking help*	*Only when pain persisted*: When the pain set in, I thought, ‘I must see about this.’ (Interview 1: 17–37).
	*Reasons for not seeking help*: Sometimes you'll say, ‘why have I not investigated’… and I think ‘stop complaining’. I think that's what you do when you're older. (Interview 1: 351–357). So I think you just don't speak about things because you don't want to be a moan, nobody's wanting to hear about somebody‘s aches and pains, so you just brush over them a bit. (Interview 1: 437–440). Their (hospital consultant) time is so precious. (Interview 2: 130).
*The importance of diagnosis*	*Increases worry*: There was an MRI scan, but again, very disappointing because I just thought ‘oh know, they'll see really where this is’, but they didn't’ they didn't see anything. (Interview 1: 95–87 & 135; repeated Interview 2: 120–125.) I imagine all my body is getting to get like this. (Interview 1: 184; repeated Interview 2: 257).
	*Invisibility of pain*: I think it would give me peace of mind knowing, because all along, there's so many of my friends haven't a clue that I've got this. (Interview 1: 370–372).
	*Feels like a fraud*: Nobody realises I'm putting up with this, I feel like a fraud. (Interview 1: 831; Interview 2: 149–150).
	*Lack of expectation*: I don't expect (GP) can do anything about it now, no nothing. (Interview 2: 211–212).
*Being listened to and being heard*	*Effort involved in presenting to HPs*: I don't sort of go on about it, I just brief tell them and that's it. (Interview 2: 207–208).
	*Being nice not enough*: He was very sympathetic, but I don't want sympathy, I don't want people to feel sorry for me. (Interview 2: 159).
	*(Different) Expectations*: (Hospital consultant) They more or less said ‘well there's no point in me having another appointment with you. You just felt ‘that's so final.’ (Interview 2: 123–124). (GP) said ‘I think you should make up your mind that you're going to have to live with it.’ (Interview 1: 64). (New Hospital Consultant) He said ‘I can see that it would mean an awful lot to you if you knew exactly where that pain was coming from.’ (Interview 1: 715–718).
	*Knowing own body*: I said (to GP), ‘I'm wondering if it's related to anything with the problem I have in my leg?’ and she just brushed passed it. (Interview 1: line 609–611; repeated Interview 2: 201–204 &210).
Notes	Sought help from GP only after experiencing back pain for a ‘couple of years’ – when pain extended down her leg. New hospital consultant recognised how important diagnosis was to her. She repeats issues around lack of diagnosis several times in both interviews. Appeared to be a conflict between not wanting to make a fuss but needing reassurance and someone to talk to about her concerns.

The example used here is Female 13, Aged 66, Caucasian, Undiagnosed back and leg pain (‘nerve pain’).

### Ethical considerations

The study was approved by the North of Scotland Research Ethics Committee (09/SO802/93). Verbal and written informed consent was obtained before participants' first and second interviews and all data were anonymised.

### Reflexivity

The team involved in the study comprises researchers from nursing, medicine, physiotherapy, occupational therapy medical and health economics backgrounds, and a practising General Practitioner (GP). The research was conducted as part of a large collaborative project on self-management for older people with pain: Engaging with Older People and their Carers to develop interventions for the self-management of Chronic Pain (EOPIC), the background to which is reported online (www.eopic@dundee.ac.uk).

## Results

Twenty-three participants (16 women, 7 men) were recruited aged from 66 to 89 years (median  = 73 years). Of these four had university education, seven had college education, and the remaining 12 did not have any further educational qualifications. Eighteen lived in the city and the remaining participants lived in rural areas. Fourteen of these participants lived alone and the remaining nine lived with their spouse/partner.

Three themes emerged: *Seeking help*; *The importance of diagnosis*; and *Being listened to and being heard*. We present findings directly relating to participants' perceptions of interactions with GPs, nurses, physiotherapists, and hospital doctors. A range of quotes from different participants (here ascribed a code, F = female, M = male) was chosen that succinctly captured a theme across interviews.

It should be noted that no differences were found between Caucasian and Chinese participants' perceptions. However, Chinese participants chose to be interviewed as a group, once only and, at times, needed the help of an interpreter to clarify our questions and help them express their answers. Answers tended to be short, probing was difficult, and they might have been reluctant to discuss personal issues in the presence of their peers [24].

### Theme: *Seeking help*

In the UK, GPs would be expected to be a particularly appropriate source of help for older adults who are frequent users of primary care [17]. Amongst participants, an insidious onset of pain seemed to contribute to delays in seeking help from GPs. Only when the pain became persistent, more noticeable and repetitive was help sought:

When the pain set in, I said, “I must see about this” (F13).

A variety of reasons were offered for participants' reluctance to see their GP, although the concern not “to bother” physicians, or “waste” their time appeared paramount. One woman, who had experienced back pain for “many years” said she needed to be “really ill” before she went to a doctor (F17). Comparisons were made to other “elderly” people; participants felt that they were “lucky” not to be as bad as them. This appeared to add to their reticence to seek professional support since they were concerned not to be seen as “complainers” by visiting GPs “too often.” For a few participants, decisions about seeking help seemed to be influenced by the belief that “nothing much” could be done about their pain since they associated it with getting older:

It's something that, as you get older, you're going to have to live with (M19).

Managing the impact of pain concomitantly with engaging with HPs seemed to consume a great deal of emotional effort and self-protection and seemed also to account for delays in seeking help. Much effort was spent thinking about how they should present to HPs, reflecting their concern not to be seen as making a “fuss.” Participants considered hard how they might present their accounts succinctly, without complaint or being over-assertive:

I try and smile, I try and be cheery I try not to be pushy [laughs]. I try to say “I'm here for help” but I leave feeling… worried (F10).

### Theme: *The importance of diagnosis*

Feelings of worry were exacerbated when no cause could be found for their pain:

If it was a known pain, I could say “well that could be so and so…” When I get a pain that I can't put a name to … I worry… (F10).

Most participants discussed the importance of having a diagnosis and the frustration when none was apparent:

“When I think about it, I think there must be some reason for this, why… you know?” (M3).

The invisibility of pain made the diagnosis particularly important. Commonly, participants worried about being seen as “a fraud” or “making it up.” One woman described encounters with various HPs and her relief when her pain was “labelled”:

I felt people thought I was making it up, making a fuss about nothing. Eventually, something showed on the x-ray. They said “of course, you've got a painful hip” And I thought, “I've been telling you that for years”. It was osteoarthritis. So I did get a label eventually (F14).

Another participant said that “the most disappointing thing” about her pain was not having a diagnosis. This led to a lack of expectation that things might improve in the future:

As far as this leg is, I don't expect him [GP] to do anything about it now, no, nothing (F13).

In contrast, an 82 year-old woman expressed hope that her pain would be cured once diagnosed:

I just keep thinking it's going to get better! I think maybe after I see this doctor, he'll find something that could be sorted (F11).

A few, however, felt it was insufficient to simply name their condition:

The general [comment from doctors that] it must be a nerve problem that cannot be explained is frustrating (F13).

One woman was unimpressed by the consultant's diagnosis of sciatica since she felt she had not had the tests to support the claim:

[He] breezed into the room like a gale of wind and said “what are you doing back here? There's nothing we can do for you!” … But I stuck to my guns and managed to extract an MRI scan for my knee (F15).

Another woman was so relieved at her diagnosis that she was unconcerned about the consultant's “blunt” approach:

There was no sort of “I'm sorry to have to give you this diagnosis or”…. The nurse was a bit taken aback. She said “are you okay? That was a bit harsh, wasn't it?” And I said “no, it's a relief to have a diagnosis” (F14).

An 81 year-old man was the exception in reflecting that the inability of doctors to give him a diagnosis for his pain was understandable given his age:

Maybe it's not their [doctors'] fault. As people get older, it's like an old car, it's breaking down a bit (M12).

### Theme: *Being listened to and being heard*

Participants described becoming anxious and confused when they perceived that HPs had been disparaging and dismissive rather than supplying the information and support anticipated. This, it was felt, led to treatment that was based on assumptions, (“professionals tend to want to make up their own minds”) rather than their own experiences and knowledge of their own bodies. Again, the importance of presentation – this time the participant's history – was emphasised:

You've to have your story very short and succinct, present it well. You've to get through to him [GP]. If he short circuits you, because he's a habit of putting his hand out to try and stop you speaking, you've got to shut up [laughs]! He does it all the time! You can never get your story out. …I've had physio three times, but it doesn't help… nobody seems to listen to me (F15).

GPs who took time to listen were perceived as “traditional” or “family practitioners” who participants felt “fortunate” to have. One woman said, “I've a holistic GP practice,’ elucidating “they really get to know the person”. She also said she was “lucky” with her orthopaedic surgeons, “they were very approachable and listened.” (F14). Another described experiencing “nothing but kindness from everyone, doctors, nurses, receptionists”. She clarified that that this was because the nurses visited her at home and spent time listening to her (F8). Simply being “nice”, however, was not welcomed if ineffective:

All the doctors are very nice, but they don't take any notice…They don't do anything (F10).

It was also apparent how participants' unmet expectations could affect the interaction:

Most [physiotherapists] are lovely girls. I went to lots of them and the doctors believe in it… I just can't. They give you a bit of elastic to pull on and chat away and say nice things, but I thought I'd get a manipulation or something (M12).

There was some ambiguity in participants' perceptions concerning whether their age affected HPs' approach to them. One woman felt aggrieved by hospital doctors talking to her daughter “over my head,” which she attributed to her age (F8). The perceived reluctance of HPs to manage pain in all but a “conservative way” was felt also by some to be influenced by age:

Is it because I'm over 65, they're not doing anything about it? (M9)

It was reflected that the study had given some the first opportunity to talk at length about their pain. One man said:

Who's asked me? The GP, her attitude is “nothing very much you can do about it” (M9).

Others went further:

I think the attitude to people over 70 is wrong, just tablets, treat them conservatively…a lot of people have got a lot of life in them still … (F15).

A few participants were unsure that HPs' approach was to do with their age, recognising that this was not always their peers' experience:

I get tablets, but [GP] wasn't willing to give me an operation. She didn't come out and say “your heart is maybe not very good” but I'd the feeling maybe that was my age [82], but a lot of people older than me get hips and knees and things. (F11).

One woman went further in suggesting that HPs sometimes made too many assumptions and should be more prepared to listen to older adults about their pain precisely because of their advanced age. She considered that older individuals are more conscious of their bodies and sensitive to when something is wrong such as pain:

Sometimes, doctors don't give people credit for knowing their bodies. We've lived in our bodies and as we get older, we've lived in them for a long time; we're aware of when things are not working as well as they should and it's sometimes difficult to convince them (F14).

## Discussion

Our description of three main themes adds to the theoretical knowledge about the interaction between older people with chronic pain and their health professionals, providing insight into the perspectives of older people with chronic pain in the community. This perspective is relatively poorly represented compared to that of older people in specialist pain services. Clinically, these findings should help inform health professionals' approaches to older adults with chronic pain, specifically with respect to communication and managing expectations.

We reported how a reluctance to be judged by health professionals as wasting their time might delay seeking help for their pain. This observation is consistent with previous studies on pain in adults of different ages and a variety of clinical settings [11], [12]. In our study, the advanced age of participants was a factor in this: rightly or wrongly there were presumptions that health professionals would be dismissive of their condition as simply a part of old age. We also observed self-judgement in this context of being reluctant to seek help, whereby people felt that they would be taking up health professionals' time that could be better used by people they viewed as more deserving of that opportunity. Their judgement appeared to involve, to some extent, suppositions that there were others worse-off than them. Again, the advanced age of participants was involved in this with opinions expressed that because of their age there was nothing that could be done to help compared to younger adults for whom a professional consultation would be more appropriate. This introduces the idea of social comparison influencing decisions to engage with health professionals.

Social comparison has been investigated to a limited extent in pain [25], [26]. Its influence appears to be complex and dependent on the specific context. In our study, such comparisons appeared to add to people's reluctance to seek help or pursue treatment. Our interpretation is that the social comparisons we witnessed were harmful in the sense that they were involved in preventing people receiving something that may have helped. However, in research with older adults attending pain clinics comparisons with others were interpreted as distracting from the individual's pain and contributing to a feeling of wellbeing [6]. The scope of our study does not allow any concrete conclusions about this but it does introduce to the field the idea that social comparison may be an important phenomenon to explore further in research and to be aware of when interacting with older people with pain.

Once engaged with HPs, our participants put much effort into the presentation of self and their pain accounts. This adds to findings from previous studies on chronic illness in general amongst mixed aged groups rather than specifically relating to older adults with chronic pain [27]. The desire to present oneself in a positive manner may involve a mix of respect for the professionals' status and a strategy to avoid being judged as unnecessarily moaning. Inadvertently, however, this positive face could distract from the underlying reason for the consultation to seek help for a situation that they are having difficulty in coping with.

In our sample the desire for a diagnosis was evident. An ongoing search for diagnosis can be associated with a biomedical “cause and cure” understanding of chronic pain [28]. Findings elsewhere have suggested that many older adults attending pain clinics accept that their chronic pain is not curable [7], [8]. This is indicative of a more biopsychosocial understanding of chronic pain compared to our sample. Education to facilitate a biopsychosocial understanding of pain is an expected feature of all but the most biomedically-focused pain clinics. Perhaps our sample, recruited from the community rather than pain clinics, had not had access to that education. Diagnosis has been identified as a frame of reference for individuals with chronic pain, clarifying and providing explanation for what they experience [29], [30]. Without this scaffolding, anxiety and worry may creep in, as highlighted by our study and other research with working and mixed age groups [31]. The clinical and research communities may be moving towards viewing chronic pain as a long-term condition in its own right, defined in biopsychosocial terms. However, our data suggest that for many older adults affected there remains a high likelihood of a belief that pain is a symptom of an underlying condition, a belief that gives rise to a strong expectation of a medical diagnosis. The challenge to clinicians is to meet this expectation of diagnosis with a clear and plausible explanation consistent with a biopsychosocial model of pain, taking care to avoid their communication being interpreted as dismissive through vague language or language that implies age as an insurmountable barrier towards management of pain.

Good practices in communication were described, primarily where professionals demonstrated empathy with the person. Empathy has been described as underpinning satisfactory and successful consultations [32], [33]. However, participants did not always feel that they were listened to or heard: physicians sometimes were perceived to have unhelpfully controlled pain communication by interrupting participants' reports of pain. This is in contrast to participants' desire for the opportunity to tell their story in sufficient detail and their antipathy towards being asked to give abbreviated descriptions of their pain and its effects [22]. Indeed, participating in our research presented some with the first opportunity to talk at length about their pain, as others have found [5], [6], [15]. It was also felt that credence was not given to older individuals' knowledge and experience of their own body. When partnership is encouraged in clinical decision-making this is, at least, a missed opportunity.

Our findings should be treated as illustrative rather than representative. The geographical location - NE Scotland - meant that possible influences and interplay of factors such as ethnicity, socio-economic circumstances or sites of pain could not be investigated in-depth. This phase of our study sought older adults' views only, so findings reflect their perspective, not the view of HPs who frequently report frustration in having inadequate time, training or resources to meet the support needs of chronic pain patients [34].

Older adults' confidence in reporting pain is likely to be increased if they are listened to and believed, if discussions about pain are actively facilitated, and their awareness of treatments and services is improved [15]. For an individualised approach to be realised, older adults' expectations about symptoms, treatment and management of their pain should be clarified, and they should be encouraged to express their concerns.
